# Hepatic β-arrestins: potential roles in liver health and disease

**DOI:** 10.1007/s11033-023-08898-0

**Published:** 2023-10-16

**Authors:** Alzahraa Muhammad Eissa, Mohamed H. Hassanin, Islam A. A. E. H. Ibrahim

**Affiliations:** 1 Faculty of Pharmacy, Zagazig University, Zagazig, 44519 Egypt; 2 Department of Pharmacology and Toxicology, Faculty of Pharmacy, Zagazig University, Zagazig, 44519 Egypt

**Keywords:** β-arrestin-1, β-arrestin-2, Hepatic disorders, Liver fibrosis, Hepatocellular carcinoma

## Abstract

Β-arrestins are intracellular scaffolding proteins that have multifaceted roles in different types of disorders. In this review article, we gave a summary about the discovery, characterization and classification of these proteins and their intracellular functions. Moreover, this review article focused on the hepatic expression of β-arrestins and their hepatocellular distribution and function in each liver cell type. Also, we showed that β-arrestins are key regulators of distinct types of hepatic disorders. On the other hand, we addressed some important points that have never been studied before regarding the role of β-arrestins in certain types of hepatic disorders which needs more research efforts to cover.

## Introduction

Arrestins, originally known as the “retinal S antigen” due to their action in uveitis, were first identified as a significant retinal photoreceptor protein component [1, 2]. In early 1980s, a research on the visual system revealed the ability of arrestins to turn off the light-activated rhodopsin [1, 3]. In 1986, after the cloning of β2-adrenergic receptor (β2AR), it was showed that the β2AR putative membrane topology was comparable to that of rhodopsin [1, 4]. Subsequent studies indicated that an arrestin-like protein was involved in the modulating of non-visual G protein-coupled receptors (GPCRs) [1, 5]. Later, two separate visual arrestins have been cloned (known as arrestin-1 and arrestin-4), as well as two non-visual arrestins (alternatively known as β-arrestin-1 or arrestin-2 and β-arrestin-2 or arrestin-3) [1, 6–8]. Further studies revealed that both GPCR kinases (GRKs) and arrestins were essential to regulate the desensitization of β2AR and the signaling function of other GPCRs [1].

β-arrestin-1 and -2 isoforms share 78% of their amino acid sequences with some coding differences such as those in the C-termination [9]. Some previous studies have shown that β-arrestin-1 and -2 are functional substitutes for each other to some extent [10]. β-arrestin-1 and -2 share the ability to interact with activated GPCRs, but they are different in terms of expression patterns, specificity for certain GPCRs, and functional effects [11, 12]. β-arrestin-1 and -2 act as scaffold proteins in different signaling pathways, however it is usually cell type and receptor specific [11]. For example, β-arrestin-2 not β-arrestin-1, is a requisite for the activation of c-Jun N-terminal kinases (JNKs) pathway [13].

### β-arrestins as desensitizing proteins

G protein-coupled receptors (GPCRs), also known as 7-transmembrane receptors (7TMRs) are the most diversified and well-represented type of membrane receptors [14]. Light, hormones and neurotransmitters are among the stimuli that GPCRs can recognize and translate into the pertinent intracellular messages [15]. Most of the biochemical reactions in the cell, cell motility, cell structure, and gene expression can all be influenced by GPCR activation [16]. GPCR signaling is involved in nearly every aspect of the human physiology, thus it’s no surprise that GPCRs are involved in around 40% of the current prescribed drugs, either directly or indirectly [17].

Receptor desensitization is the process in which frequent stimulation of the GPCRs leads to lower response within seconds to minutes [18]. Down-regulation, on the other hand, is the process of decreasing signaling within hours [18]. The dissociation of Gα and Gβγ subunits caused by receptor-dependent activation of heterotrimeric G-proteins promotes their interactions with effector proteins, which results in downstream signaling [11, 19]. Most agonist-activated GPCRs are rapidly uncoupled from G-proteins leading to signal desensitization through a process of two steps that includes: GRKs phosphorylation and β-arrestins binding [20]. While the physical binding of β-arrestins hinders the G-protein coupling to GPCR, there are other mechanisms for β-arrestins to ensure that G-protein signaling is effectively blocked [16]. β-arrestins scaffold cyclic nucleotide phosphodiesterases (PDEs) and also diacylglycerol kinases (DGKs) which degrade second messengers downstream of the activated receptors [16, 17]. Additionally, β-arrestins function as linker proteins, allowing active GPCRs to be removed from the cell surface, a process known as GPCR internalization. Receptor-associated β-arrestins bind to clathrin and clathrin adaptor protein (AP2) directly to promote GPCR internalization through clathrin-coated pits [21, 22].

### β-arrestins as scaffolding proteins

Over the last 20 years, many studies have indicated the role of β-arrestins as scaffolding proteins in signal transduction. β-arrestins can regulate the activity of cellular Src (c-Src), the three well-known mitogen-activated protein kinase (MAPK) pathways, cyclic adenosine monophosphate (cAMP) phosphodiesterase, protein phosphatases, ubiquitin ligases, calmodulin, deubiquitinating enzymes, and a lot of other signaling proteins [23, 24]. Although the activity of many β-arrestins seems to necessitate binding with the activated GPCRs, it has now been shown that β-arrestins can regulate cellular signaling in a GPCRs-independent manner. For example, it has been proven by in vitro studies that β-arrestin-2 can activate JNK3 without any of the GPCRs, whereas β-arrestin-stimulated activation of extracellular signal-regulated protein kinases 1 and 2 (ERK1/2) normally needed the interaction with GPCRs [23–25]. In addition, β-arrestins, especially β-arrestin-1 not β-arrestin-2, have been found, at least under specific experimental conditions, to be present in the nucleus, on which they can alter the patterns of gene expression [21, 26]. These results show that β-arrestins also have a role in regulation of many nuclear and cytoplasmic functions.

### Liver physiology and functions

The liver is a vital organ in the human body that performs a variety of functions including bile secretion, minerals and vitamins storage, drug metabolism, bilirubin metabolism, glucose synthesis and metabolism, protein and amino acid synthesis and metabolism, lipid and cholesterol synthesis and metabolism, metabolic detoxification, immunological functions, and vascular and hematologic functions as it acts as a vital blood reservoir [27, 28]. It is also involved in the thyroid hormone function since it is the site where T4 is deiodinated to T3 [29]. In addition, the liver is responsible for the production of almost all body plasma proteins, such as binding globulins, albumin, protein C, protein S, as well as all intrinsic and extrinsic pathways of clotting factors [29]. Liver is considered a unique organ because it has a dual blood supply from both the portal vein (75%) and the hepatic artery (25%) [29].

The portal vein carries blood from the gastrointestinal tract, spleen, pancreas and gallbladder to the liver. In the liver, portal vein ramifies and reaches the sinusoids, where blood is directed to the central vein at the hepatic lobule level, then to the hepatic veins and inferior vena cava, then to the systemic venous system [30]. The lobule is the functional unit of the liver. It is hexagonal in shape, with a portal triad of portal vein, hepatic artery, and bile duct at each side [28, 29].

Notably, liver produces bile which travels through a series of ducts and exits through the common hepatic duct into the gallbladder, where it is concentrated and stored. Contraction of gallbladder pushes bile through the cystic duct again into the common bile duct. Then bile enters the duodenal lumen. In the small intestine, bile acids facilitate lipid digestion and absorption. About 5% of bile acids are then excreted while most bile acids are reabsorbed from the ileum, secreted into the portal venous system, and returned to the liver. This process is known as enterohepatic recirculation [31].

The liver is made up of different cell types such as hepatocytes, Kupffer cells, hepatic stellate cells (HSCs), liver sinusoidal endothelial cells and biliary epithelial cells (cholangiocytes) [27]. Each one of these cell types has its own set of functions that work together to maintain hepatic functions on numerous levels. Hepatocytes are the liver’s main epithelial cell type. They account for most of the volume of the liver and fulfill many of liver functions [27]. Kupffer cells are the liver's resident macrophage cell type. They can recognize the multiple pathologic stimuli brought via the portal circulation and depending on various contributing circumstances, can perform pro-inflammatory or anti-inflammatory functions in the healing process of liver wounds [27]. HSCs are a dynamic cell type that can be in either a quiescent or active state. Their function is vitamin A storage in lipid droplets in their quiescent state, but their further roles in this state are still unknown [27]. The activation of stellate cells occurs when the liver is damaged, resulting in their proliferation and gradual loss of their vitamin A content [27]. HSCs can also control collagen deposition and organization in wounded livers, contributing to liver scarring that can lead to cirrhosis, a serious condition that may lead to end-stage liver disease [27]. Liver sinusoidal endothelial cells are specialized type of endothelial cells that have unique features [27]. Eventually, cholangiocytes are the second most prevalent epithelial cell type in the liver and serve as the cells that line the bile duct lumen [27].

### Hepatic expression of β-arrestins and their signaling pathways

#### Expression and signaling in hepatocytes

Hepatocytes play a leading function in the regulation of glucose and lipid metabolism throughout the body. Hepatocytes express a large amount of the Gs-coupled glucagon receptors (GCGRs). Hormone-activated GCGRs initiate a series of cAMP/protein kinase (PKA)-dependent processes that enhance gluconeogenesis and glycogenolysis, resulting in a significant rise in hepatic glucose production (HGP) which is a key feature of type 2 diabetes [22]. Hepatic β-arrestin-2 is essential to maintain the normal concentration of glucose in the blood. In vivo and in vitro, β-arrestin-2 acts as a powerful negative regulator of hepatic GCGR signaling at the cellular level [32]. In contrast to β-arrestin-2, hepatic β-arrestin-1 does not appear to be involved in the regulation of hepatic GCGRs activity and euglycemia [32]

#### Expression and signaling in Kupffer cells

Kupffer cells are the main players in lipopolysaccharide (LPS)-induced liver injury [33]. Endogenous LPSs originated from the intestine were found to activate Kupffer cells which enhance the production of pro-inflammatory cytokines as: tumor necrosis factor (TNF)-α, interleukin (IL)-1β, and IL-6 which could exacerbate the liver damage [34]. Jiang et al. have found that the deletion of β-arrestin-2 in mice increased Kupffer cells production of pro-inflammatory cytokines such as TNF-α, IL1β, IL-6 and IL-10, aggravating LPS-induced liver damage [35]. Moreover, this pathway may also play a role in Toll-like receptor 4/Nuclear Factor Kappa B (TLR4/NF-κB)-mediated inflammation [35].

#### Expression and signaling in HSCs

HSCs are the predominant liver fibrogenic cell type. HSCs activation is critical for fibrosis development. This activation includes the trans-differentiation of HSCs from a quiescent state to myofibroblast-like cells, with the development of α-smooth muscle actin (α-SMA) and the loss of cellular storage of vitamin A [36]. Accelerated proliferation and increased synthesis of extracellular matrix (ECM) components distinguish the activated HSCs [37]. β-arrestins expression was found to be related to HSCs proliferation in an experimental hepatic fibrosis. As hepatic fibrosis progressed, the β-arrestin-2, not -1 expression increased gradually [38]. So, the depletion of β-arrestin-2 in HSCs inhibits HSCs mitogenic signaling and proliferation by decreasing the platelet derived growth factor-BB (PDGF-BB)-induced activation of the ERK1/2 pathway which means that targeting β-arrestin-2 could be beneficial in the treatment of liver fibrosis [38]. β-arrestin-2 may also enhance NADPH oxidase 4 (NOX4) expression and reactive oxygen species (ROS) which are significant factors in liver fibrosis via ERK and JNK signaling [39]. In addition, the in vitro decreased β-arrestin-2 has shown to suppress both formation of HSC collagen and increased expression of type III transforming growth factor-β1(TGF-β1) receptor (TRIII), and also downregulating the Smad2, Smad3 and Akt which are the components of TGF-β1 pathway [40].

#### Expression and signaling in sinusoidal cells

Sinusoidal endothelial cells (SECs), the specialized endothelial cells that act as a barrier between both hepatocytes and hepatic stellate and blood cells, have an important role in regulation of all liver functions [41]. SECs represent a permeable barrier which is considered the most permeable endothelial cells of the mammalian body with the highest endocytosis capacity. They regulate the hepatic vascular tone contributing to the maintenance of a low portal pressure [39]. Endothelial nitric oxide synthase (eNOS), which is expressed in hepatic SECs, mainly regulates endothelial functions, vascular tone and hepatic blood flow by producing nitric oxide (NO) in the liver [42]. G protein-coupled receptor kinase-interacting protein 1 (GIT1) plays a positive role in GPCR-mediated eNOS signaling as it stimulates the enzymatic activity of eNOS [43]. Endothelin-1 (ET-1) signaling stimulates Src kinase tyrosine phosphorylation of GIT, resulting in activation of eNOS/NO [44]. In liver, β-arrestins-2 is primarily expressed in SECs and acts as a key component of the GPCR–eNOS signaling pathway which enhances eNOS activation, whereas β-arrestins-1 is mainly found in hepatocytes [45]. In SECs, ET-1 was found to promote β-arrestins-2 and eNOS colocalization and β-arrestin-2 overexpression was found to promote GIT1–eNOS interaction which lead Liu et al. to show that ET-1-induced GIT1 tyrosine phosphorylation is β-arrestin-2-dependent, indicating that ET-1 signaling to GIT1–eNOS to promote eNOS activation is mediated by β-arrestins-2-Src kinase complex [45]. In addition, β-arrestin-2 expression appeared to decrease after liver injury, resulting in disruption of the GIT1/eNOS/ NO signaling [45]. So, β-arrestin-2 downregulation in injured liver SECs leads to decreased NO generation and portal hypertension in that condition [45].

#### Expression and signaling in cholangiocytes

Cholangiocytes are the epithelial cells that line the intra and extrahepatic bile ducts and play a major role in determining the bile composition and flow [46]. Bile acid signaling in cholangiocytes is carried out by G-protein-coupled bile acid receptor, Gpbar1 (TGR5), a GPCR located in the cilium, apical plasma membrane, and subapical compartment [47]. TGR5 activation promotes distinct variations in the cAMP and ERK levels in ciliated and non-ciliated cholangiocytes. In non-ciliated cholangiocytes, TGR5 agonists enhance cAMP levels and decrease ERK signaling, promoting proliferation. TGR5 agonists, on the other hand, limit proliferation of ciliated cholangiocytes via decreasing cAMP levels and increasing ERK signaling [48, 49]. This variation in TGR5 agonists effects appeared to be as a result of binding of TGR5 to Gα_s_ protein in the non-ciliated cells and Gα_i_ protein in the ciliated ones [48, 49]. TGR5 was found to be a unique GPCR which doesn’t interact with β-arrestin-1 or 2 or GRK2, 5 or 6 but signals through plasma membrane rafts when activated with its endogenous agonists [50].

**Fig. 1:**
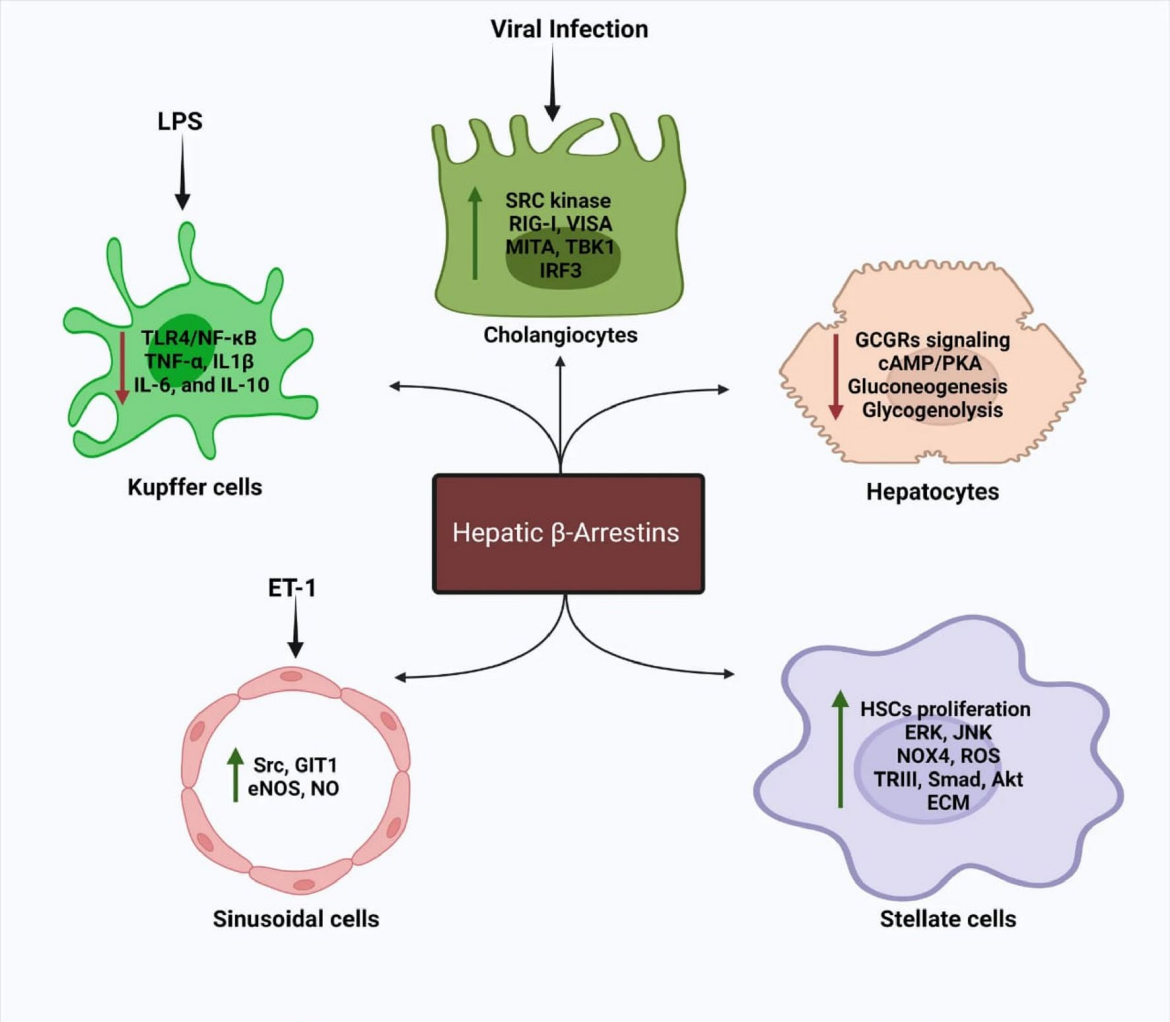
A graphical summary of β-arrestins signaling in the different types of hepatic cells. LPS lipopolysaccharides, TLR4 toll like receptor 4, NF-κB nuclear factor κB, TNF-α tumor necrosis factor α, IL-1β,-6,-10 interleukin-1β,-6, 10, RIG-I retinoic acid-inducible gene I, VISA virus-induced signaling adaptor, MITA mediator of interferon regulatory transcription factor 3 activation, TBK1 TANK binding kinase 1, IRF3 interferon regulatory factor 3, GCGR Gs-coupled glucagon receptor, cAMP Cyclic adenosine monophosphate, PKA protein kinase A, GIT1 G-protein coupled receptor kinase-interacting protein 1, eNOS endothelial nitric oxide synthase, HSC hepatic stellate cells, ERK extracellular regulating kinase JNK c-Jun N-terminal kinase, NOX4 NADPH oxidase 4, ROS reactive oxygen species, TRIII type 3 transforming growth factor β1, Akt protein kinase B, ECM extracellular matrix. Upward arrows refer to increased expression or activity of the listed proteins. Downward arrows refer to decreased expression or activity of the listed proteins

In contrast to the activity of endogenous bile acids (BAs), a synthetic agonist, 3-(2-chlorophenyl)-N-(4-chlorophenyl)-N, 5-dimethylisoxazole-4-carboxamide (CCDC), can stimulate TGR5 interaction with β-arrestin-2 and GRK2. Only CCDC at high concentrations has been shown to induce a small signal between β-arrestin2, GRK2 and TGR5, and no signal between β-arrestin1 or GRK5 or 6 and TGR5 [50]. Hu et al. have also shown that viral infection promotes an increase in intracellular BAs, resulting in activation of Src kinase through the TGR5-GRK-β-arrestin axis, that modulates tyrosine phosphorylation of various antiviral signaling components such as retinoic acid-inducible gene I (RIG-I), virus-induced signaling adaptor (VISA), mediator of IFN regulatory transcription factor 3 activation (MITA), TANK binding kinase 1 (TBK1) and interferon regulatory factor 3 (IRF3) in order to enable the innate antiviral immunity [51]. A graphical summary of β-arrestins signaling in the different types of hepatic cells is shown in Fig. 1**.**

### Hepatic immune disorders and role of β-arrestins

#### 1ry biliary cholangitis

Primary biliary cholangitis (PBC), previously known as primary biliary cirrhosis, is an autoimmune liver disorder distinguished by female prevalence, severe lymphocytic cholangitis, particular anti-mitochondrial antibodies (AMAs), and autoreactive T-cells that destroy the primary bile ducts [52, 53]. β-arrestins are critical for T-cell survival [53, 54]. The expression of β-arrestin-1 was observed to be considerably higher in T-cells from PBC patients [53]. Furthermore, in PBC patients, the level of β-arrestin-1 mRNA was positively linked with the Mayo risk score [53]. Overexpression of β-arrestin-1 was found to increase proliferation of T-cells and production of interferons, downregulate NF-κB and activator protein 1 (AP-1) activities, and promote histone H4 acetylation in the CD40L, LIGHT, IL-17 and interferon-γ promoter regions, while downregulate H4 acetylation in the TRAIL, Apo2 and HDAC7A promoter regions, just like that modulating these genes expression [53]. So, it is suggested that β-arrestin-1 plays a vital regulatory role in PBC patients' autoreactive T-cells and contributes to the disease's pathogenesis, which has important implications for innovative therapeutic strategies and adds to our understanding of autoimmune disease mechanisms [53].

### Hepatic cancer and tumors and role of β-arrestins

#### Hepatocellular carcinoma

Hepatocellular carcinoma (HCC) is the most common type of primary liver cancer. HCC occurs most often in people with chronic liver diseases, such as cirrhosis which can be caused by viral hepatitis B or C infection. Also, non-alcoholic steatohepatitis associated with metabolic syndrome or diabetes mellitus is becoming a more common risk factor of HCC in the West [55]. Notably, β-arrestins have been found to play an important role in HCC by controlling cell proliferation, invasion, migration, angiogenesis, and metastasis. These effects are thought to be mediated via various signaling pathways, including MAPK/ERK, Wnt/β-catenin, NF-κB, and phosphoinositide-3 kinase (PI3K)/Akt pathways [56]. In the same context, it has been found that β-arrestin-2 level, but not β-arrestin-1, decreases in patients with HCC especially in malignant cells compared with normal ones. In addition, β-arrestin-2 overexpression has been found to reduce cell migration and invasion in cultured HCC cells and this overexpression inhibits Akt activation. Conversely, β-arrestin-1 accelerates metastasis in HCC through phosphorylation of ERK1/2 and its effect on the expression of epithelial-mesenchymal transition (EMT) markers. Furthermore, the HCC-associated inflammation increases TNF-α production that directly induces hepatic β-arrestin-1 expression which then mediates Akt phosphorylation, resulting in malignant proliferation of liver cells [57]. Therefore, targeting β-arrestins may represent a potential therapeutic strategy of HCC [58].

### Chronic hepatitis and role of β-arrestins

#### Alcohol-induced hepatitis

Alcohol-induced hepatitis or alcoholic liver disease (ALD) is a complicated disease that became one of the leading causes of severe liver-dysfunction [59]. ALD includes a wide range of hepatic lesions, from steatosis to cirrhosis, and even hepatocellular carcinoma. The metabolic products of ethanol are highly toxic and can induce hepatocellular apoptosis and necrosis. These toxic effects are thought to be mediated by increased β-arrestin-2 expression. In-vivo and in-vitro studies have shown that β-arrestin-2 knockdown inhibits hepatocyte apoptosis, whereas over-expression of β-arrestin-2 induces apoptosis in ALD by suppressing Akt signaling [60].

#### Non-alcoholic steatohepatitis

Nonalcoholic steatohepatitis (NASH) is a type of liver inflammation and damage caused by lipid metabolism dysregulation and fat accumulation in the liver. NASH occurs in people who don't abuse alcohol. Notably, decreased β-arrestin-1 expression has been observed in patients with NASH which may accelerate steatohepatitis development. On the contrary, upregulation of β-arrestin-1 level has been found to alleviate NASH-associated pathological changes, and this effect can be amplified in the presence of overexpressed pro-growth differentiation factor 15 (pro-GDF15). Remarkably, β-arrestin-1 has been found to interact with growth differentiation factor 15 (GDF15) and facilitate the transportation of its precursor (pro-GDF15) to the Golgi apparatus for cleavage and maturation [61].

#### Liver fibrosis and role of β-arrestins

Liver fibrosis is the excessive deposition of ECM proteins, six times more than normal, that causes chronic damage to the liver. It is considered a wound-healing response to chronic liver injury. Several causes of hepatic injury such as viruses, alcohol abuse, metabolic syndrome and cholestasis produce mediators that induce inflammation in hepatocytes and biliary cells. Damaged hepatocytes and biliary cells release inflammatory cytokines that activate Kupffer cells and stimulate the recruitment of activated T-cells. These activated cells stimulate the activation of resident HSCs which transdifferentiate into myofibroblast-like cells, acquiring contractile, proinflammatory, and fibrogenic properties. Stimulated HSCs also secrete cytokines that keep them activated. The accumulation of activated HSCs leads to increased synthesis of ECM proteins, resulting in tissue fibrosis [62]. ECM proteins include collagens (I, III and IV), fibronectin, undulin, elastin, laminin, hyaluronan, and proteoglycans [62].

The expression of hepatic β-arrestin-2 increases in human liver fibrosis. It has been found that, there is a strong association of β-arrestin-2 expression, the activation of HSCs, and the overexpression of collagen 1 and α-SMA [63]. In the same context, β-arrestin-1 can activate HSCs by stimulating autophagy-mediated snail or mannan-binding lectin serine protease 1 (MASP1) signaling leading to liver fibrosis [64, 65]. β-Arrestins have a crucial role in the activation of G-protein-independent signaling pathways like the Hedgehog, Wnt, Notch and TGF-β pathways, as well as downstream kinases such as MAPK and PI3K. These signaling pathways play a potential role in the pathogenesis of fibrosis and fibrotic diseases [66].

#### TGF-β signaling pathway

The TGF-β signaling pathway has a crucial role in regulating various physiological and pathophysiological functions, including cell proliferation, apoptosis, differentiation, inhibition of tumor cell migration and the development of cancer and fibrosis [55]. TGF- β1 is the most abundant isoform in the liver and is secreted by stellate cells. Following liver injury, HSCs become activated and secrete latent TGF-β, which forms an autocrine positive feedback loop that promotes fibrogenesis via Smad2/3 [67]. In addition, several Smad-independent pathways, such as MAPKs and PI3K/Akt can be activated by TGF-β signaling and contribute to progression of liver fibrosis [68].

Despite that some studies showed that β-arrestin2 can induce liver fibrosis by activating the TGF-β pathway, other studies showed the reverse. It has been found that β-arrestin-2 in some situations can negatively regulate TGFβ signaling and increase the endocytosis of the TGF-β receptor [69]. It seems that the β-arrestin-2 mediated signaling is multifaceted and is affected by several factors.

#### PI3K/Akt signaling pathway

**Fig. 2:**
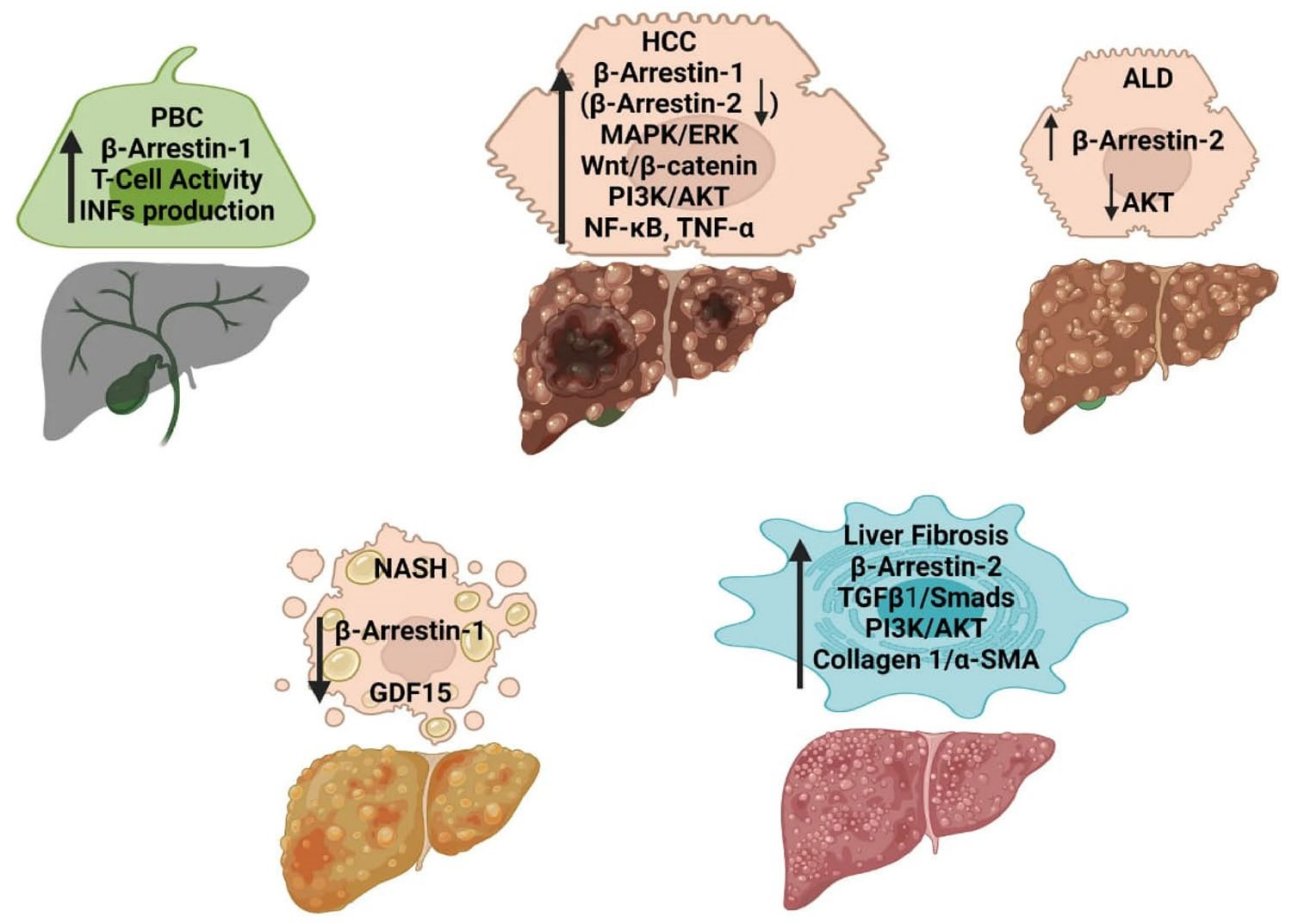
A Graphical summary of the roles of β-arrestins in hepatic disorders. PBC primary biliary cholangitis, INF interferon, HCC hepatocellular carcinoma, MAPK mitogen activated protein kinase, ERK extracellular regulating kinase, PI3K phosphatidyl inositol 3 kinase, Akt protein kinase B, NF-κB: nuclear factor Κb, TNF-α tumor necrosis factor α, ALD alcoholic liver disease, α-SMA α smooth muscle actin, NASH non-alcoholic steatohepatitis, GDF15 growth differentiation factor 15. Upward arrows refer to increased expression or activity of the listed proteins. Downward arrows refer to decreased expression or activity of the listed proteins

Like TGF-β signaling, PI3K/Akt signaling pathway is involved in several pathologic conditions including fibrosis. Activated PI3K/Akt pathway regulates cell survival, cell cycle progression, and cell growth. On the other hand, inhibition of PI3K/Akt pathway can induce HSC apoptosis, inhibit ECM deposition, including synthesis of type I collagen, and reduces expression of profibrogenic factors [70]. Also, β-arrestins can both activate or inhibit the PI3K/Akt pathway confirming the multifaceted roles of β-arrestins. β-arrestins can mediate Src interaction with Akt promoting activation of the later. On the other hand, the protease activated receptor 2 (PAR-2) can inhibit the activation of PI3K through a β-arrestin-dependent pathway [66]. A graphical summary of the roles of β-arrestins in the different types of liver disorders is shown in Fig. 2**.**

### Liver regeneration after ischemic insults or partial resection and role of β-arrestins

Ischemia-reperfusion injury (IRI) is a pathological process characterized by increased cellular dysfunction and death, following return of blood flow to a previously ischemic tissue [71]. Liver IRI is common among patients undergoing liver transplantation, partial hepatectomy, or after circulatory collapse. Notably, knockdown of hepatic β-arrestin-2 inhibits the activation of PI3K/Akt pathway promoting hepatocyte apoptosis and impairs their ability to proliferate leading to worsening of liver IRI [72]. In the same context, hepatectomy or liver resection is a surgical operation by which a part of or total liver is removed. If a part of liver (up to 70% of its original volume) is removed, it can grow back to its former size due to its high regeneration capacity [73]. Interestingly, preconditioning with remifentanil, a potent short-acting opioid analgesic, has been found to enhance liver regeneration. Furthermore, remifentanil can protect hepatic tissues from ischemia and reperfusion injury through anti-inflammatory effects. Notably, the ability of remifentanil to enhance liver regeneration is mediated by supporting hepatocyte proliferation and by increasing β-arrestin-2 expression with subsequent upregulation of the ERK/cyclin D1 pathway [74].

## Conclusion and future perspectives

Β-arrestins are important intracellular scaffolding proteins that have multifaceted roles in different types of hepatic diseases making them promising targets for identification of new treatments of these disorders. β-arrestins are expressed in all types of hepatic cells and can perform distinct functions in each type. Moreover, β-arrestins have been found to play a potential role in either progression or regression of certain types of hepatic disorders. On the other hand, this review article revealed some types of hepatic disorders at which the role of β-arrestins have never been investigated such as autoimmune hepatitis, 1ry sclerosing cholangitis, bile duct cancer, liver cell adenoma, hemochromatosis, hyperoxaluria, Wilson’s disease, α1-anti-trypsin deficiency, and acetaminophen hepatotoxicity. Furthermore, and despite that β-arrestins can potentially modulate distinct types of hepatic disorders, there are no clinically approved pharmacotherapies that can protect the liver by targeting the β-arrestin pathway. Therefore, more efforts are needed to cover these points.

## Data Availability

Not applicable.
